# Book Reviews in Brief

**Published:** 1913-01-25

**Authors:** 


### Book Reviews in Brief.

Notes on the Treatment of Tuberculosis. By John Laird, L.R.C.S.I., L.R.C.P.I. (Bristol: J. Wright and Sons. 1912. Pp. 85. Price 2s. 6d. net.)

The author of this brief volume is a firm believer in the virtues of a particular prescription as a curative agent for all forms of tuberculosis. He does not decry sanatoria and other hygienic measures, but thinks that drug treatment has been too much neglected. His prescription is of the "shot-gun" variety; and although the modern school of scientific clinicians is prone to sneer at "poly-pharmacy," Mr. Laird is undeterred by little things of that sort. The prescription itself contains in a dose: half to one grain of sodium iodide; five to ten grains of sodium benzoate; half to one minim of Fowler's solution; one to two drachms of tincture of pulsatilla; and three to five drops of tincture of baptisia. Neither of the latter drugs is contained in the British Pharmacopœia, and we have only Mr. Laird's word for their sovereign effects. Still it is vastly to his credit that he should frankly and fully profess the faith that is in him, instead of adopting the methods of the proprietors of secret remedies. We cannot honestly say that he has made out a case for his pet prescription; but at any rate he has adhered to the traditions of the profession by publishing it, and it is now open to anyone to make a trial of it and issue an account of the results.

Elementary Clinical Pathology for Nurses. By George Herschell, M.D., and Richard Weiss, Ph.D. Second Edition. (London: J. and A. Churchill. 1913. Price 1s.)

Despite a few blemishes, this inexpensive little book contains definite and clear instruction on the performance of those tests generally required in the examination of the urine, stools, and vomit, and also describes how the nurse may assist the doctor in his preparations for examining the blood and throat swabbings. There are many things in this book which are not usually regarded as falling in the province of the nurse, but, as the authors explain, the nurse in charge of a case in private has more responsibilities than in a hospital, where dressers and house surgeons are available. The examination of the urine takes up about two-thirds of the book; the tests given are not always those most commonly employed, and indeed there are indications that this book is meant to be used with a special set of apparatus, which is duly advertised at the end of the book! We should strongly demur to the suggestion on page 8 that some antiseptic ought to be added to the urine to prevent decomposition even in a twenty-four hours' collection. It is useless to state that the specific gravity taken at a temperature other than 60° F. would be incorrect if it is not also mentioned how this error can be corrected. On page 16 an error is left in this second edition, making 3 per cent. equal to 3 grammes per litre. A curious misuse of the word "clinical" occurs in the heading of the chapter relating to taking a swabbing of the fauces.

Pathfinders in Medicine. By Victor Robinson. (*Medical Review of Reviews*, 206 Broadway, New York. Price 10s. net.)

This book is a series of essays on a number of medical men who, in the author's opinion, may justifiably be considered as pioneers in medicine. It is somewhat difficult to class the book, which is essentially hybrid, though unfortunately not in the original classical sense of the term. To readers who know anything at all about the history of medicine there is not a single new fact or observation in all these papers. Moreover, the inflated, redundant method of expression which the author favours is as annoying as the amuck enthusiasm which pervades certain chapters. As an instance of both faults we may cite the article on Semmelweiss. The reader who is familiar with the history of the Vienna school will at once know where the author's historical details are incorrect; those, again, who have read Markusovsky's biography of Semmelweiss in the *Orvosi Hetilap,* or even Sinclair's appreciative—and not too scholarly—Life, will hardly thank Mr. Robinson for trying to vision the pioneer of antiseptic surgery in a halo of purple patches. Some of these azulean touches are amusing, but both the author and Dr. Jacobi, who sponsors the volume in an introduction, appear to be sublimely unconscious of this humour. As a contribution to serious historical literature the book is not worth consideration; as a means of bringing home to the layman, or even to the doctor who knows nothing of the history of his art, some of the facts in connection with the evolution of the principles of medicine and surgery it has some points in its favour. But in our opinion these merits are considerably overshadowed by the absurd manner in which the essays are phrased and by the comparatively high price at which the volume is sold. A popular, journalistic book like this should be catalogued at a figure not exceeding half-a-crown.

